# Pharmacy Practice and Education in Croatia

**DOI:** 10.3390/pharmacy6030089

**Published:** 2018-08-21

**Authors:** Marijana Zovko Končić, Jeffrey Atkinson

**Affiliations:** 1 Faculty of Pharmacy and Biochemistry, University of Zagreb, A. Kovačića 1, 10000 Zagreb, Croatia; mzovko@pharma.hr; 2 Pharmacolor Consultants Nancy, 12 rue de Versigny, 54600 Villers, France

**Keywords:** pharmacy, education, practice, Croatia, European Union

## Abstract

The PHARMINE (“*Pharmacy Education in Europe*”) project examined the organisation of pharmacy practice and education in the European Union (EU). An electronic survey was sent out to community, hospital, and industrial pharmacists, and university staff and students. This paper presents the results of the PHARMINE survey for Croatia. We examined to what extent harmonisation with EU norms has occurred, whether this has promoted mobility, and what impact it has had on healthcare.

## 1. Introduction

The project PHARMINE (“*Pharmacy Education in Europe*”) surveyed pharmacy practice and education in the member states of the EU, including Croatia, between 2008 and 2011, with an update in 2017. An overview of the methodology used has already been published [1]. PHARMINE gathered information on general, community practice and specialised hospital and industrial practice. PHARMINE also included assistant pharmacists.

PHARMINE studied the administration of pharmacy practice and education, which, in the EU, fall under two jurisdictions: national and European. The latter involves confederal decision-making. The freedom of the right to work anywhere in the EU is one of the key premises. For pharmacy and other sectoral professions, such as medical doctors, this right is regulated by automatic recognition of professional qualifications by member states. The EU issues directives or ordinances laying down the broad imperatives [2]. An EU directive requires member states to achieve a particular result, without dictating the exact means of achieving that result, thus allowing leeway to organise systems that are more or less harmonised. Member states may introduce national legislation relating to specialisation in practice and education, to ownership and management of pharmacies, etc.

In parallel to the EU directive above, pharmacy education is impacted by the Bologna agreement on the harmonisation of degree courses and student exchange [3]. The Bologna agreement was signed by the education ministers of the countries of the European Higher Education Area (48, including the 28 EU member states). In contrast to the EU directive, Bologna recommendations are not legally binding. Recommendations include a degree structure with a first cycle—bachelor’s degree (3 years)—followed by a second cycle with a master’s degree (2 years) and a doctorate. Such a system promotes professional mobility, with an early passage from a short university career (3 years) to employment. This is in contradiction to the EU directive that requires a 5-year, “tunnel” degree structure for pharmacy, i.e., a degree course that offers no possibility for intermediate mobility/employment after the accomplishment of a 3-year bachelor’s period. 

As seen in the previous paragraph, mobility is the driving principle of the Bologna declaration and is behind other aspects of the Bologna Process, such as the development of tools to promote student exchange, such as the European Credit Transfer and Accumulation System (ECTS) [3]. ECTSs are given for defined learning outcomes and their associated workload, and they are laid down in a Diploma Supplement (DS) describing the nature, level, context, content, and status of the studies. ECTSs coupled to the DS allow students to validate studies carried out at the host university by their home university. This paper looks at how the principles of the Bologna Process described above have developed in Croatian universities. It is particularly interesting to examine how this affects pharmacy practice and education in a country that recently joined the EU; this occurred during the last enlargement of the EU in 2013, following the Croatian declaration of independence in 1991 and diplomatic recognition of Croatia by the European Economic Community in 1992. 

In order to place pharmacy practice within the general health context in Croatia compared to the EU, it can be noted that in Croatia, life expectancy at birth (Table 1) is lower than (males) or similar to (females) the EU average of 79 years. Healthy life expectancy (EU average 68 years) is similar. Expenditure on health is approximately one-half of the EU average ($3611 per capita). 

## 2. Design

Information was obtained on:

- Pharmacy practice:
  Community, hospital, and industrial;
  Organisation;
  Legislation;
  Education and training;
- The adoption of the EU sectoral directive 2013/55/EU [2];
- The impact of the Bologna declaration [3]:
  Organisation of the degree structure;
  Implementation of the ECTS and the DS;
  Student exchange under the ERASMUS (EuRopean Action Scheme for the Mobility of University Students, [6]) or other schemes.

The information is presented in the form of tables in order to facilitate legibility. This form of presentation was developed in association with this journal’s editorial board and has been described in detail in a previous publication [7].

## 3. Evaluation and Assessment

### 3.1. Organisation of the Activities of Pharmacists, Professional Bodies

Table 2 provides details of the numbers and activities of community pharmacists and pharmacies in Croatia. Items are expounded in the “comments” column, which also includes opinions and trends for the future.

The number of inhabitants per pharmacist is half the EU average, whereas the number of number of inhabitants per pharmacy is similar to the EU norm. This means that there are more pharmacists per pharmacy in Croatia compared to the EU average. The activities and occupations of pharmacists in Croatia are similar to those of community pharmacists in other EU member states [1]. Globally, as far as pharmacy practice is concerned, Croatia is close to or even above EU norms and in line with the EU directive 2013/55/EU [2], thus, pharmacy practice is globally harmonised with that in the other member states, although expenditure on health is half the European average (Table 1). 

It should be noted that, in contrast to other papers in this series, the comparison with the linear regression index for the EU, which takes into account the population of the country (see [1] for details), cannot be used here, as the reference paper [1] was published (in 2011) before Croatia acceded to the EU.

Table 3 provides details of the numbers and activities of persons other than pharmacists working in pharmacies in Croatia.

Table 4 provides details of the numbers and activities of hospital pharmacists in Croatia.

Croatian hospital pharmacists have similar activities and responsibilities to those elsewhere in the EU [1]. The hospital section of the Croatian Pharmaceutical Society is a member of the European Association of Hospital Pharmacists.

Table 5 provides details of the numbers and activities of industrial pharmacists and pharmacists in other sectors in Croatia.

In Croatia, industrial pharmacists and pharmacists working in other sectors have similar practices and duties to those in other EU countries [1]. As accurate numbers of industrial pharmacists and pharmacists working in other sectors were not available for most European countries, a comparison with the EU average is not possible. Croatian industrial pharmacy is characterised by a great deal activity in generic products. 

Table 6 provides information on professional associations for pharmacists in Croatia.

Administrative and other procedures in Croatia are similar to those elsewhere in the EU [1]. One special aspect is the obligation to follow continuing education programmes in order to retain one’s license.

### 3.2. Pharmacy Faculties, Students, and Courses

Table 7 provides details of pharmacy higher education institutions (HEIs), staff, and students in Croatia.

Pharmacy studies are multidisciplinary; they include basic (mathematics, chemistry, physics, statistics, biochemistry, biology, molecular biology), biomedical (anatomy, physiology, pathophysiology, microbiology and parasitology, pharmacology, toxicology, and haematology), and pharmaceutical courses (pharmaceutical botany, pharmacognosy, pharmaceutical chemistry, biochemistry of drugs, pharmaceutics, drug design, drug analytics, clinical pharmacy, pharmacotherapy, cosmetology, etc.), through which students acquire specific knowledge and skills in the field of pharmacy.

Table 8 outlines the specialisation electives in Croatia.

Specialisation courses last for two semesters, during which students collect 60 ECTS points, of which 40 are through 10 core subjects and 20 are through elective courses. At the end of the course, a thesis paper is written. The specialist studies are approved by the University of Zagreb. By defending the final specialist thesis, the participants acquire the academic title University Master of a given area (drug development, medicine biochemistry and laboratory medicine, dermopharmacy and cosmetology, clinical pharmacy, molecular diagnostics, or phytopharmacy with dietotherapy). The abbreviation for all the academic titles is *univ. mag. pharm*. 

It is important to note that the postgraduate specialist study in Clinical Pharmacy is also a part of a 3-year specialisation in Clinical Pharmacy, approved by the Croatian Ministry of Health. Besides the teachers from the Faculty of Pharmacy and Biochemistry, clinical pharmacy specialists, as well as experienced hospital pharmacists, are involved in this course. Comparing this information with Table 4, it is seen that “hospital” pharmacists are defined more by their place of work and duties than by their qualifications, whereas “clinical” pharmacists are defined more by their qualifications. It is expected in the future that these two definitions will merge as more and more clinical pharmacists are recruited into hospital pharmacies. 

Table 9 provides details of past and present changes in pharmacy education and training in Croatia.

### 3.3. Teaching and Learning Methods

Table 10 provides details of hours by learning method (for further details on the definitions of the different methods, see Reference [1]), exemplified by Faculty of Pharmacy and Biochemistry, University of Zagreb—the largest institution dedicated to the education of pharmacists. 

Almost 40% of the time is spent on lectures, whereas traineeship occupies 21% and only 4% is spent on project work. In the middle and final years, there are several electives offered (9 in the third year, 10 in the fourth, and 8 in the fifth), thus allowing students to individualise their degrees to some extent.

### 3.4. Subject Areas

Table 11 provides details of student hours by subject area (for further details on the definitions of the subject areas, see Reference [1]) at Faculty of Pharmacy and Biochemistry, University of Zagreb. Student hours are presence hours, not student workload hours.

The MEDISCI/CHEMSCI ratio (955/885 = 1.1) reveals a course that is balanced in terms of “scientific” and “medicinal” subjects, as is the case elsewhere in the EU [1]. “Basic” subjects (CHEMSCI, PHYSMATH, BIOLSCI) are concentrated in the early years, whereas more “applied” subjects (MEDSIC, PHARMTECH) are studied in the later years. Traineeship is essentially in the fifth year. Such chronological harmonisation is similar to that observed in other EU member states and should facilitate student exchange.

### 3.5. Impact of the Bologna Recommendations [3]

Table 12 provides details of the various ways in which the Bologna declaration impacts on the pharmacy HEIs of Croatia.

The numbers of places open for mobility through Erasmus are indicated. The actual exchange may vary (usually it is significantly lower). Although there are 46 places for outgoing students, usually less than 10 apply for these scholarships.

### 3.6. Impact of EU Directive 2013/55/EC [2]

Table 13 provides details the various ways in which the EC directive impacts on pharmacy education and training in Croatia.

Croatia mainly conforms to the different aspects of the EU directive with a notable “tunnel” degree. In spite of harmonisation with the rest of the EU, as seen in Table 12, this does not assure full student mobility due to obstacles other than the study program.

## 4. Discussion and Conclusions

The survey reveals that community pharmacy practice in Croatia is similar to that elsewhere in the EU. One specificity of practice in Croatia is that the license to practice is delivered and maintained on the basis of a genuine commitment to continuous professional development.

There is a certain degree of specialisation in pharmacy practice. Firstly, we consider industrial pharmacy. The pharmaceutical industry is centred on generics and distribution. EFPIA figures for 2015 reveal that Croatia had the lowest investment in pharmaceutical R&D in the EU at 40 M€, and that the pharmaceutical trade balance is negative at −241 M€ [12]. This situation is associated with a lack of teaching in the area of industrial pharmacy. Specialisation in hospital or clinical pharmacy is starting to develop. Although the hospital pharmacist is defined at the present by place of work and responsibilities rather than by education and training, recently, a course in clinical pharmacy has opened at Zagreb University. As more and more such clinical pharmacists are employed in hospital pharmacies, the practice of the hospital pharmacist will improve and become more specialised. 

Pharmacy education and training in Croatia follow the EU model, with several harmonising features, such as:

- A balance between “scientific” (chemical and other subjects) and “medicinal” subjects (such as pharmacology, etc.);
- An evolution throughout the degree course from “basic” subjects (physical, sciences, mathematics, etc.) in the early years to more applied subjects, such as pharmaceutical technology and medicinal sciences, in the later years;
- Traineeship in the fifth year.

Although on the whole the course in education and training is well harmonised with the EU model and directive, this is not the case for the Bologna principles. This is reflected in student exchange, which remains low compared to the number of places available.

Regarding the final aspect of the effect of pharmacy practice and education on healthcare in Croatia, some positive effects can be noted. For example, the number of inhabitants per pharmacist is low compared to the EU average, and the number of pharmacists per pharmacy is higher. Still, life expectancy in males is lower than the EU average. One major factor here is the need for a general increase in healthcare expenditure, currently running at half the EU average. Other elements could possibly play a role, such as the development of pharmaceutical care and the role of pharmacists in vaccination campaigns and public health actions, like blood pressure and sugar measurement, and anti-smoking campaigns. 

## Figures and Tables

**Table 1:** Health statistics for Croatia [4,5].

Total Population	4,240,000 (2015)
Life expectancy at birth, m/f (years)	75/81
Healthy life expectancy at birth, m/f (years)	67/72
Total expenditure on health per capita	$1652

**Table 2:** Numbers and activities of community pharmacists and pharmacies in Croatia.

Item	Numbers	Comments
Pharmacists	3705	1144 inhabitants/community pharmacist. EU average 2145 [1].
Pharmacies	1130	Inhabitants/pharmacy: 3752. EU average 4407 [1]. Pharmacists/pharmacy: 3.3. EU average 2.1 [1].
Competences and roles of community pharmacists		Care for the welfare of the patient in all circumstances; Guidance and advice to patients on proper use and monitoring of drug effects; Taking care of supply of drugs, medical devices, and other means to protect health; Managing medicines for some specific ailments; Contribution to rational and economic prescribing of drugs.
Is ownership of a community pharmacy limited to pharmacists?	No	The owner of a private, independent, community pharmacy can be only pharmacist. A private community pharmacy chain consists of at least two community pharmacies; the founder of the chain can be a physical or legal entity.
Rules on geographical distribution of pharmacies?	Yes	To obtain permission, each pharmacy needs to meet conditions defined by the “*Ordinance on criteria for defining the area where pharmacy will be opened*” of the Croatian government: Communities of up to 3000 insured persons: 1 pharmacy; Communities of 3000–8000 insured persons: 2 pharmacies; For every supplementary pharmacy: additional 5000 insured persons.
Are drugs and healthcare products available to the general public by channels other than pharmacies?	Yes	Prescription drugs are sold in pharmacies. Non-prescription drugs are classified as: BR = non-prescription drugs sold only in pharmacies; BRX = non-prescription drugs on the “General Sale List” that can be sold in pharmacies and specialised stores for retail sale of medical devices and medicinal products (drugstores). Food supplements can be sold also in cosmetic shops, so-called “specialized shops”, “herbal drugstores”, and other shops.

**Table 3:** Numbers and activities of other personnel working in pharmacies in Croatia.

Item	Numbers	Comments
Are persons other than pharmacists involved in community practice?	Yes	They are designated as “pharmaceutical technicians”. These are technicians, not students.
Their numbers and status	1–2 per pharmacy	
Organisations providing and validating education and training of the 3-year courses		School for pharmaceutical technicians.
Subject areas		Anatomy, physiology, fundamentals of the health profession; general, organic, and analytical chemistry; biochemistry; pharmaceutical chemistry; pharmacology; medical microbiology; botany with pharmacognosy; pharmaceutical technology with cosmetology; food chemistry; natural remedies; the industrial production of medicines; introduction to laboratory work.
Competences and roles		Dispensing and counselling of products under the control of a pharmacist.

**Table 4:** Numbers and activities of hospital pharmacists in Croatia.

Item	Numbers	Comments
Does such a function exist?	Yes	The function is defined by government in the Croatian Law on Pharmacy [8].
Number of hospital pharmacists	90	See the website of the Croatian Pharmacy Society [9] and that of the Croatian Pharmaceutical Society—Hospital Pharmacy Section [10].
Number of hospital pharmacies	49	See the website of the Croatian Pharmacy Society [9].
Competences and roles of hospital pharmacists		Part of the multidisciplinary patient-care team. Provision of a supply of hospital healthcare facilities, with medication and medical products involving: Purchasing and packaging (unit-dose drug distribution) of drugs and medical material; Preparing galenical (obtained from plant or animal tissue) and magistral (made up according to a special prescription) preparations necessary for hospital activities; Monitoring of drug use.

**Table 5:** Numbers and activities of industrial pharmacists and pharmacists in other sectors in Croatia.

Pharmaceutical and Related Industries (Alphabetical Order)		
Item	Number	Comment
Number of companies with production, R&D, and distribution	3	Belupo (http://www.belupo.hr/) Jadran Galenski Laboratorij (JGL) http://www.jgl.hr PLIVA Hrvatska, member of the TEVA group http://www.pliva.hr/ See [11].
Number of companies with production only	3	Genera http://www.genera.hr/ PharmaS http://www.pharmas.hr/ Yasenka http://yasenka.hr/ See [11].
Number of companies with distribution only	4	Medical Intertrade http://www.medical-intertrade.hr/ Medika www.medika.hr Oktal pharma http://www.oktal-pharma.hr Phoenix farmacija www.phoenix-farmacija.hr See [11].
Number of companies producing generic drugs only	5	Belupo (http://www.belupo.hr/ Genera http://www.genera.hr/ Jadran Galenski Laboratorij http://www.jgl.hr PharmaS http://www.pharmas.hr/ PLIVA Hrvatska http://www.pliva.hr/ See [11].
Industrial pharmacy		
Number of pharmacists working in industry	10%	Approximately 10% of pharmacists in Croatia
Competences and roles of industrial pharmacists		Research and development, formulation, production, quality assurance, pharmacovigilance
Pharmacists working in other sectors		
Number of pharmacists working in other sectors	10%	Approximately 10% of pharmacists in Croatia
Sectors in which pharmacists are employed		Regulatory, education, marketing

**Table 6:** Professional associations for pharmacists in Croatia [8,9].

Roles of Professional Associations		
Item	Yes/No	Comments
Registration of pharmacists	Yes	There is legal obligation for pharmacists working in community and hospital pharmacy to be registered (licensed) in Croatia. Pharmacists are obliged to follow continuing education programmes in order to keep their licence for independent work. Licence renewal occurs every 6 years and necessitates following professional continuing education programs or passing an examination at the Croatian Chamber of Pharmacists.
Ethical and other aspects of professional conduct	Yes	Ethical and other aspects of professional conduct are described in “*Kodeks ljekarničke etike i deontologije*” (Ethical Codex), published in 1996, and the document “*Pravila dobre ljekarničke prakse*” (Good pharmacy practice guidelines) published in 1997 [9].
Quality assurance and validation of higher education institution (HEI) courses for pharmacists	Yes for continuing education.	The *Commission for continuing professional education of pharmacists* at *Croatian Chamber of Pharmacists* makes decisions regarding the verification and evaluation of the courses and other forms of continuing education of pharmacists.

**Table 7:** Pharmacy higher education institutions (HEIs), staff, and students in Croatia.

Item	Number	Comments	
Total number of HEIs in Croatia	1 + 1	Faculty of pharmacy: Faculty of Pharmacy and Biochemistry University of Zagreb (Faculty of pharmacy) www.pharma.hr Integrated undergraduate and graduate university study programme, pharmacy, University of Split School of Medicine http://www.mefst.unist.hr/studies/other-integrated-studies-in-croatian/pharmacy/2238	
Public	2		
Organisation of HEIs			
		Zagreb	Split
		Independent faculty	Attached to the medical faculty
Teaching staff			
		Zagreb	Split
Teaching staff		89	271
Full-time employees		64	158
Partial time employees		25	113
Professionals from outside the HEIs		110 (mostly student supervisors during the traineeship in the pharmacies)	40 (mostly student supervisors during the traineeship in the pharmacies)
Students			
		Zagreb	Split
Entry places		140	30
Number of applicants for entry		450 (3–3.5 applicants/place)	105 (3.5 applicants/place)
Graduates that become registered pharmacists		135	27
Number of international students		5	0
Entry requirements			
		Zagreb	Split
Specific pharmacy entrance examination		No	No
Advanced entry		Depends on the year	Depends on the year
Fees per year			
For home and EU students		Free of charge for first year, later depends on the success of the student	Free of charge for first year, later depends on the success of the student
For non-EU students		20,000 Kn (approx. 2700 €)	10,000 Kn (approx. 1350 €)

**Table 8:** Specialisation electives in pharmacy HEIs in Croatia.

Course	ECTS	Fees EU/Other Nationalities	HEI
Clinical Pharmacy	60	2700 €/3400 €	Zagreb
Dermopharmacy and cosmetology	60	2700 €/3400 €	Zagreb
Drug development	60	2700 €/3400 €	Zagreb
Phytopharmacy with Nutrition Therapy	60	2700 €/3400 €	Zagreb
Medical biochemistry and laboratory medicine	60	2700 €/3400 €	Zagreb
Molecular diagnostics	60	2700 €/3400 €	Zagreb

**Table 9:** Past and present changes in education and training in Croatian pharmacy HEIs.

Item	Comments
Have there been any major changes since 1990?	The programme has been changed three times. The latest programme includes 6 months’ professional training, as required by the EU directive [2].
Are any major changes envisaged before 2019?	No

**Table 10:** Student hours by learning method *.

Method	Year 1	Year 2	Year 3	Year 4	Year 5	Total-Electives	%
Courses							
Lecture	315	360	330 (+electives)	300 (+electives)	75 (+electives)	1380	37
Tutorial	165	115	115 (+electives)	90 (+electives)	60 (+electives)	545	15
Practical	180	210	210 (+electives)	195 (+electives)	30 (+electives)	825	22
Project work					150	150	4
Traineeship							
Community			30	60		90	2
Community and hospital					720	720	19
Total-electives	660	685	685	645	1135	3710	100

* Faculty of Pharmacy and Biochemistry, University of Zagreb.

**Table 11:** Student hours by subject area *.

Subject Area	Year 1	Year 2	Year 3	Year 4	Year 5	Total (-Electives)	%
CHEMSCI	285	300	165	135		885	25.0
PHYSMATH	150					150	4.2
BIOLSCI	150	100	75			325	9.2
PHARMTECH			105	150	60	315	8.9
MEDISCI		285	310	300	60	955	26.9
LAWSOC	75				15	90	2.5
GENERIC					15	15	0.4
GENERIC + traineeship			30	60	735	825	23.3
Total (-electives)	660	685	685	645	870	3545	100.0

* Faculty of Pharmacy and Biochemistry, University of Zagreb. The abbreviations used are: CHEMSOC: chemical sciences; PHYSMATH: physical and mathematical sciences; BIOLSCI: biological sciences; PHARMTECH: pharmaceutical technology; MEDISCI: medicinal sciences; LAWSOC: law and social sciences; GENERIC: generic competences.

**Table 12:** Ways in which the Bologna declaration impacts on Croatian pharmacy HEIs *.

Bologna Principle	Is the Principle Applied?	Comments
Comparable degrees/Diploma Supplement (DS)	Y	DS in English: On demand
Two main cycles (B and M) with entry and exit at B level	N	
ECTS system of credits/links to LLL	Y	Students with 300 ECTSs or more can continue their education at Faculty of Pharmacy and Biochemistry (e.g., do their Ph.D.)
Obstacles to mobility	Y	Mobility is strongly promoted, but language, finances, lodging, and other things unrelated to the Faculty may be potential obstacles to the students who wish to study abroad.
European QA	Y	Coordinated by the Agency for Science and Higher Education
ERASMUS staff exchange to Croatian HEI from elsewhere		Places open for mobility: 32
ERASMUS staff exchange from Croatian HEI to other HEIs		Places open for mobility: 33
ERASMUS student exchange to Croatian HEI from elsewhere		Places open for mobility: 45
ERASMUS student exchange from Croatian HEI to other HEIs		Places open for mobility: 46

* Example: Faculty of Pharmacy and Biochemistry, University of Zagreb.

**Table 13:** Ways in which the elements of the EC directive (left column) impact on Croatian pharmacy HEIs *.

Item	Comments
“Evidence of formal qualifications as a pharmacist shall attest to training of at least five years’ duration…”	The duration of study is 5 years.
“…four years of full-time theoretical and practical training at a university or at a higher institute of a level recognised as equivalent, or under the supervision of a university”	Five years of full-time theoretical and practical training is provided.
“…six-month traineeship in a pharmacy which is open to the public or in a hospital, under the supervision of that hospital's pharmaceutical department.”	Six-month traineeship in a pharmacy—which is open to the public or in a hospital, under the supervision of that hospital's pharmaceutical department—is provided.
“The balance between theoretical and practical training shall, in respect of each subject, give sufficient importance to theory to maintain the university character of the training.”	The balance between theoretical and practical training gives sufficient importance to theory to maintain the university character of the training.

* Example: Faculty of Pharmacy and Biochemistry, University of Zagreb.
